# Postoperative enterocutaneous fistulas: Management outcomes in 23 consecutive patients

**DOI:** 10.1016/j.amsu.2021.102413

**Published:** 2021-05-19

**Authors:** Ibrahim Falih Noori

**Affiliations:** Department of Surgery, College of Medicine, University of Basrah, Iraq

**Keywords:** Enterocutaneous fistula, Post-operative, SOWATS protocol, Management

## Abstract

**Background:**

About 75%–85% of enterocutaneous fistulas are postoperative. Such fistulas are still disastrous and frustrating complication for surgeon and the patient and their management remains one of major challenge and dilemma in general surgical practice. Strict adherence to treatment guideline according to SOWATS protocol could results in better outcomes.

**The objective:**

of this study was to assess the management outcomes of 23 patients with postoperative enterocutaneous fistulas.

**Methods:**

A total of 23 consecutive patients with postoperative enterocutaneous fistulas during the period of study were included. These fistulas were classified anatomically and physiologically. The predictive factors for spontaneous closure, management outcomes and mortality rate factors for were studied.

**Results:**

Overall Closure of the fistulas was accomplished in 16 patients (69.6%). Spontaneous closure after successful conservative management was seen in 4 patients (17.4%). The average time between fistula development and spontaneous closure was 32 days (range12–66 days). Of 19 patients underwent corrective surgery, closure was achieved in 12 patients (52.2%), thus the surgical success rate was 63.2%. Surgical intervention was performed after an average period of 28 days (range 18–42 days) from diagnosis of the fistula. Five patients with high output (21.7%) died because of sepsis, severe malnutrition and organ failure.

**Conclusion:**

adherence to standardized protocol and multidisciplinary approach of patients with enterocutaneous fistulas could results in good outcomes. A reasonable period of conservative treatment is always required to optimize patient's general conditions and for spontaneous closure of fistula. Operative correction is usually required for proximal high output and complex fistulas. The complexity of the fistulas, sepsis, high output effluent and comorbidities are the main factors affecting healing rates and fistula related mortality.

## Highlights

• Post-operative ECF fistula is one of challenging and frustrating postoperative complication.
• Strict adherence to SOWATS protocol and multidisciplinary approach could results in better outcomes.
• A reasonable period of conservative treatment is crucial to optimized patients general conditions.
• Operative correction is usually required for proximal high output and complex fistulas.

## 1 Introduction

Enterocutaneous fistula is considered as one of major dilemma and a challenge in the general surgical practice. About 75%–85% of enterocutaneous fistulas are iatrogenic in origin which occur as postoperative complication while in 15%–25% of cases develop due to abdominal trauma or spontaneously due to malignancy, inflammatory bowel disease mainly Crohn's diseases, radiation enteritis, ischemia or infective conditions like diverticulitis and appendicitis [1,2].

Despite advances in the management of these fistula with the better control of sepsis by new and powerful antibiotics, improvement of the intensive care, nutritional status and advent of new imaging modality, the mortality rate is still high with range between 10% and 30% or even more in some series [2,3].

Enterocutaneous fistulas are commonly caused by unintended technical errors such as unnoticed enterotomy, anastomosis disruption due to excessive tension or compromised blood supply [4]. Erosion of the bowel wall by foreign body such as tube drain or by nearby abscesses can also lead to formation such fistulas [4,5]^.^Once the enterocutaneous fistula is discovered and diagnosed, the principal aim of management is to promote spontaneous closure of fistula and when this is not possible, is to resect the fistula tract and to restore the continuity of the bowel if it is possible after optimization the clinical conditions of the patient [6]. In this research we present the clinical presentation, management, and the prognosis of 23 postoperative enterocutaneous fistulas with thorough review of literatures.

## 2 Methods

This is a retrospective controlled study conducted in major hospital in Basra province, Iraq for the period between March 2014 and July 2020, in which 23 consecutive patients with postoperative enterocutaneous fistulas of different etiologies had been enrolled. They were 13 males and 10 females with age ranges between 32 and 72 years (mean 52.6 years).Patients with esophageal, gastric, pancreatic and biliary fistulas were excluded. Clinical assessment including thorough history and careful clinical examination were done for all patients. (Table 1). Informed written consent was obtained from all participants. Clinical examination includes vital signs, pallor, dehydration and nutritional status. Abdominal examination looking for the laparotomy wound, site and, degree of fistula activity assessed by inspection of wound dressings which usually stained were recorded. The type, color and amount (rate) of fistulas effluent and abdominal wall defect was also recorded.Peri-fistulous skin was carefully examined for any evidence of hyperemia, laceration or skin loss. Postoperative enterocutaneous fistulas were then classified according to their anatomy and output. Complete blood count, C - reactive protein, serum albumin and plasma transferrin were done for all patients.

**Table 1:** Primary surgery that results in enterocutaneous fistulas formation.

Initial surgery that resulted in ECF	Number	%
Perforated duodenal ulcer	3	13
Recurrent incisional hernia	3	13
Adhesive intestinal obstruction	4	17.5
Left hemicolectomy	2	8.8
Total abdominal hysterectomy and salpingectomy	3	13
Ruptured ectopic pregnancy	2	8.8
Abdominoplasty	3	13
Appendicectomy	1	4.3
Total gastrectomy	1	4.3
Gastric bypass procedure	1	4.3

**Table 2:** Characteristics of 23 patients with postoperative enterocutaneous fistulas.

Variables	Number	%
Patients number	23	1oo
Sex Male Female	13 10	56.5 43.5
Age < 60 years ≥ 60 years	8 15	34.8 65.2
Primary disease Benign Malignancy	19 4	82.6 17.4
Anatomy of ECF* Small intestine Large intestine	20 3	87 13
Output of fistula (ml/day) Mild (<200) Moderate (200–500) High (>500)	5 7 11	21.8 30.4 47.8
Serum Albumin < 25 mg/dl > 25 mg/dl	15 8	65.2 34.8
Plasma Transferrin < 250 mg/dl >250 mg/dl	13 10	56.5 43.5
Sepsis Absent Present	9 14	39.1 60.9
Skin excoriation Present Absent	12 11	52.2 47.8
Abdominal wall and laparotomy wound Intact Open (defect)	13 10	56.5 43.5
Fistulous tract Simple Complex	11 12	47.8 52.2
Number of external fistulous opening Single Multiple	18 5	78.2 21.8
Nutrition Enteral TPN Mixed	9 6 8	39.1 26.1 34.8
Co-morbidity Absent Present	14 9	60.9 39.1

Patients variables were recorded, including age, sex, initial surgery that resulted in enterocutaneous fistula, anatomical classifications (small or large intestine fistulas), output of the fistulas (low, moderate or high), presence of sepsis, fluid and electrolytes disturbances, nutrition status and albumin concentration. Abdominal wall status whether intact or open and peri-fistulous skin status were also recorded.

Patients were submitted to guided treatment according to SOWATS protocol which stands for Sepsis control, Optimization of nutritional status, Wound and skin care, delineation of fistula Anatomy, Timing of surgery and Surgical Planning and strategy.

### 2.1 Conservative management

All patients were initially treated conservatively for a period ranges from 6 to 12 weeks with the aim to enhance spontaneous closure or to perform surgery in a well and optimized conditions.

Fluid correction and replacement was done using glucose saline, Ringer lactate or normal saline using the urine output of a minimum 0.5 ml/kg/hour as a sensitive indicator of adequate tissue perfusion. Rehydration of the patients was accompanied by correction of electrolytes disturbances in particularly, hypokalaemia, hyponatraemia and metabolic acidosis.

Total enteral nutrition alone was administered to 9 patients for a period ranged 15–72 days (median of36 days) using nasoduodenal or nasojejunal feeding tubes, 8 patients with high output fistulas received total parenteral nutrition consisting of 75 g glucose, 20 g amino acids and 30 g lipids per litter and 6 patients received enteral nutrition in combination with parenteral feeding. Additional supplementation with vitamin B12, folic acid and copper was also given. Patients were allowed to ingest clear fluids up to 500 ml/day.

Sepsis was diagnosed in 14 patients by presence of fever, leukocytosis, neutrophilia, elevated C-reactive protein, decreased albumin concentration or organ failure in combination with documented infection by imaging such as ultrasound and CT scan. Drainage of abscess under imaging guidance was done when possible for 11patients. Antibiotics using parenteral third generation cephalosporin (ceftriaxone 1 g twice daily) and metronidazole 500 mg in 20 ml infusion were prescribed. Exploration when needed was considered to drain any abscess collection (3 patients).

Containment of fistula effluent and peri-fistula skin care was instituted soon as the diagnosis of enterocutanous fistula was made to decrease local skin excoriation and inflammation and prevent maceration and skin loss. For a low output fistulas, a skin barrier with a gauze dressing or occasionally pouch were used. For high output fistulas, a transparent ostomy one piece pouch was used to collect and visualize the enteric effluent. Various skin barriers were used to protect peri fistulous skin such as pectin based powders or wafers, zinc paste or cream, and spray. Patients with proximal high output fistulas, the fistula tract is intubated with a sump drain to drain excessive fluid.Ooctreotide, long-acting somatostatin analogue, proton pump inhibitors and loperamide were administrated to decrease the effluent of high output and proximal fistulas (12patients).

Enhanced computed tomography was done for 17 patients to assess the state and length of intestine, localization of fistula origin, and to look for any stenosis or obstruction and for any abscess collection. Preoperative enhanced CT scan was also taken for those patients whose their fistulas failed to close after 4–6 weeks of conservative measures (16 patients) for defining fistula anatomy. Fisulography using methylene blue ingestion either orally or through nasogastic or nasoduedenal tube was performed in 7 patients. Contrast enemas and magnetic resonance enterography were another adjuncts used to define fistulas anatomy preoperatively, (5 patients).

### 2.2 Surgical management

Nineteen patients with complex and high output fistulas failed to close spontaneously after 6–8 weeks of conservative treatment, and thus presented for corrective surgery. They were optimized clinically and psychologically to undergo corrective surgery. The hemoglobin level was≥ 10 g/dl and albumin ≥25 g/L.The sepsis was controlled and their nutrition, fluid and electrolytes disturbances were corrected prior to surgery. Their coagulation profile was also checked. One-stage repair was the main aim for patients. The procedures were performed in major private well equipped hospital by a specialist consultant surgeon of more than 15 year experience and included careful adhesiolysis, wedge excision or resection of involved segments of the intestine with limit number of anastomoses to minimum, cover sutures with healthy viable omentum or absorbable mesh insertion to keep away from anastomosis or compromised area. The median length of hospital stay was 32.6 days (range 14–75 days).

The enterocutaneous fistulas were deemed closed when there was no further communications between the bowel and abdominal wall or the laparotomy wound and no effluent or sign of inflammation are observed after 6–12 months follow up period.

The work is fully compliant with the STROCSS criteria. (www.strocssguideline.com) [7] and was registered at Researcregistry: http://www.researchregistry.com. Registration ID: researchregistry6717.

## 3 Results

A total of 23 consecutive patients presented with postoperative enterocutaneous fistulas between March 2014 and July 2020 were enrolled in this study. The median age of the patients was 53 (range 23–72) years. Thirteen (56.5%) patients were males and 10 (43.5%) were females. Postoperative fistulas appeared after a median of 18 days (range4–80 days) from initial different surgery. (Table 1).Patient's characteristics and demographics are illustrated in Table (2).All patients have been managed according to the SOWATS guideline as mentioned in the methods. Ileum was the most common site for fistulas accounting for 43.5% (10 patients). Other sites and their distribution are shown in Table (3).

Skin excoriation, cellulites, and skin loss were observed in 12 patients (43.5%). Proximal high output (duodenal) and ileal fistulas were the cause of skin excoriation in the majority of the cases (10, 83.3%). The association between skin lesion and fistula output was significant (p > 0.05).

Sepsis was detected in 14 (60.7%) patients, indicating the presence of ECF. Intra-abdominal abscess was diagnosed by CT scan in 11 of this patient. Drainage under imaging (ultrasound and CT scan) was done for these 11 patients, whereas the other 3 patients required operative drainage. Sepsis was controlled in 11 patients. The other 3 patients were died due to disseminated sepsis and organ failure mainly renal failure despite CT-guided (one patient) and operative drainage (two patients). Besides, 5 patients were found to have an intra-abdominal abscess without any sign of sepsis. Drainage under imaging guidance was done for 3 of them and open drainage was done for the rest 2 patients.

Total enteral nutrition alone was administered to 9 patients for a period ranged 15–72 days (median of36 days), 8 patients received total parenteral nutrition and 6 patients received enteral nutrition in combination with parenteral feeding.

The use of Octreotide was limited to patients with high output fistulas (12 Patients). Reduction in fistula output was observed in 8 of them (66.7%). The association between the use of Octreotide and fistula healing or decrease mortality were not observed in this open wound and abdominal wall defect were noted in 12 (52.2%) patients. The wound complications and abdominal wall defect resulted from surgical sites infection, wound dehiscence after surgery due to bowel distension and paralytic ileus and iatrogenic opening of the laparotomy wound to drain deep seated abscess and to decrease intra-abdominal pressure and prevent abdominal compartment syndrome. Ostomy transparent one piece pouch with drainable clip or fistula bag was used to collect high output fistula fluid in 13 patients. A drain inserted directly into the fistula tract to remove the moderate output fistula fluid in 6 patients. Gauze dressing with skin barrier was used to collect fistula fluid in four patients with low output and intact abdominal wall.

Overall Closure of the fistulas was accomplished in 16 patients (69.6%). Spontaneous closure after successful conservative management was seen in 4 patients (17.4%). (Table 3, Table 5).The average time between fistula development and spontaneous closure was 32 days (range12–66 days). Of 19 patients underwent corrective surgery, closure was achieved in 12 patients (52.2%), thus the surgical success rate was 63.2%. Surgical intervention was performed after an average period of 28 days (range 18–42 days) from diagnosis of the fistula. (Table 4). Five patients with high output (21.7%) died because of sepsis, severe malnutrition and organ failure, one during conservative treatment and 4 patients following surgery. Tow (8.7%) patients developed persistent fistulas after corrective and restorative surgery and a decision made to refrain from further surgery because of advanced malignancy.

**Table 3:** Enterocutaneous fistula outcomes according to location of the fistula.

Origin	No. of patients (23 patients)	Spontaneous closure (No.4) Number %	Operative closure (No.12) Number %	Total closure (No.16)) Number %	Deaths (No.5) Number %
Duodenum	5	0	2	2	3
Jejunum	4	1	2	3	1
Ileum	10	2	6	8	1^a^
Appendix	1	0	1	1	0
Colon	3	1	1	2	0^a^

a Tow patients (ileal and colonic fistulas) developed persistent fistulas after corrective and restorative surgery and a decision made to be refrained from further surgery because of advanced malignancy and inoperability.

**Table 4:** Time intervals (average) for spontaneous closure and post-operative treatment.

Origin	No. of patients	Diagnosis- spontaneous closure (days)	No. of patients	Diagnosis—operative treatment (days)
Duodenum	0	–	2	15
Jejunum	1	28	3	32
Ileum	2	15	6	22
Appendix	0	–	1	15
Colon	1	24	1	14

**Table 5:** Treatment outcomes of 23 patients with enterocutaneous fistulas according to abdominal wall status.

	Closed abdomen (11) No. %	Abdominal wall defect (12) No. %	Total population No. %
Overall closure Conservative Surgical	7 63.7 3 27.3 4 36.4	9 75 1 8.3 8 66.7	16 69.6 4 17.4 12 52.2
Success of conservative treatment	23.1	8.3	4 17.4
Success of surgical treatment	30.8	66.7	12 63.2
Morbidity persistent &recurrent fistula)	1	1	2 8.7
Overall Mortality Conservative Surgical	1 0 1	4 1 3	5 21.7 1 4.3 4 17.4

The average hospital stay in this study was 32.6 days (range 14–75 days). It was significantly longer in patients with high output fistulas compared with those of moderate and low output fistulas (43.5 days and 26.2 days respectively).

## 4 Discussion

Management of patient with enterocutaneous fistula is still represent one of major challenge and dilemma in general surgical practice [8].Although the management of such fistulas have been improved with better understanding of the anatomical,pathophysiological and metabolic derangement, better control sepsis, adequate nutritional support, and improvement in the critical care and the surgical treatment; the mortality rate is still high [[9], [10], [11]]The main concern in the treatment of enterocutaneous fistula is to enhance the spontaneous closure [10]. Although spontaneous closure occurs in some patients most of the cases need definite surgical intervention to excise the fistulous tract and to restore the continuity of intestine if the clinical and operative conditions permit [[10], [11], [12]].

The present study shows that adherence to a treatment guideline results in good general outcome. We found that optimization of patient's general conditions results in increased spontaneous closure and the recurrence rate and mortality did not influence significantly over prolonged time of conservative treatment. Similar findings are shown by Ruben GJ ET al [13].Furthermore; delay surgery and improving patient's health status improve surgical outcomes by increasing closure rate and decrease recurrence [11,14].

Proximal fistulas (duodenal and jejuneal) are indolent and aggressive and unlikely to close spontaneously than distally located fistulas (ileum and colon) (Table 6). Proximal fistulas result in marked fluid and electrolytes deficiency, malnutrition, and sepsis 15.

**Table 6:** Several variables were used clinically to judge the likelihood of spontaneous closure.

Factors	Favorable	Unfavorable
Anatomy of fistula	Colonic, ileum	Duodenum, proximal jejunum
Output	Low (<200 ml/day)	High (>500ml/.day)
Sepsis	Absent	Present
Nutritional status	Well-nourished	Malnourished
Fistula character	End fistula, long tract fistula, end fistula and small defect fistula	Short tract fistula, large defect fistula a
Intestinal continuity	Present	Absent
Serum albumin	>25 g/l	<25 g/l
Transferrin	>200 mg/dl	<200mgLdl
Distal obstruction	Absent	Present
Diseased bowel	Absent	Present
Previous abdominal; and pelvic irradiation	Absent	Present
Miscellaneous	Initial surgery at same hospital	Referred from other hospital

Serum albumin levels and plasma transferrin provide good nutritional markers [4,16].Surgery should be delayed until their levels are normalized [17,18].Serum transferrin could be useful in predicting which patients with enterocutaneous fistulas should precede to surgery despite anatomic features favorable for spontaneous closure [16,19].In this study, most patients with low an albumin and transferring were normalized prior surgery except in 6 patient when surgery was deemed necessary to prevent further deterioration. Level below 25 g/l underwent operation. These patients represented a seriously ill group that continued to show signs of inflammation despite all endeavors to treat infection and in which postponement of surgery was thought to lead to further deterioration.

Proximal small bowel fistulas have a less probability of spontaneous closure, about 20–30% (average 25%) with prolonged courses of treatment (45–70 days, average 50 days), compared to distal small bowel and colonic fistulas (75–90%, average 80%, 21–40days, 25 days respectively.) [10,20]. the rate and time elapsed between diagnosis of the fistula and spontaneous closure or operative treatment for various origins in our study are shown in Table [4].

Because spontaneous closure of enterocutaneous fistulas after conservative treatment depend on many variables, a range, varies from 15% to 70% was reported in literatures [15,21]in our study 17.4% of the fistulas healed spontaneously. We reported low rate of spontaneous closure for proximal small bowel fistulas; it recorded in only 3 of 23 patients (13.1%).Spontaneous closure of ECF did not occur after 7 weeks.

Control of sepsis and fistula effluent and are essential for spontaneous closure. We found, Spontaneous closure rate of fistulas with sepsis was significantly less than those without sepsis (4.3% versus 13%, respectively). Fluid and electrolytes loss from proximal high output fistulas can be decreased with administrations of the long acting somatostatin analogue octreotide, proton pump inhibitors and loperamide. The use of octreotide was reported to decrease fistula output, but whether it promotes or decreases the time for spontaneous closure remains a controversial issue and needs to be determined [22,23]. Recent studies found that, although output of enterocutaneous fistulas has been decreased by these hormones, results from randomized controlled studies have not including them in the standard management of enterocutaneous fistula [24].We prescribed octreotide for 16 patients proximal high output fistulas. Diminished out-put was observed in 12 (52.2%) patients.

About 70–85% of patients with enterocutaneous fistulas require operative correction in spite of initial conservative trial [21, 25].In our series, surgery was done for 19 patients. The main indication for surgery was abscess drainage and persistent fistula after at least 4–6 weeks conservative treatment. Other indications for surgery include fistulas with feature preclude spontaneous closure, such as proximal high output fistulas, large defect, end type fistula, complex multiple fistula, distal obstruction and recurrent fistulas [18,25,26].several studies [14,23,27]have shown that better outcomes were reported in patients who waited on conservative treatment for long period (6–12 weeks or even more) while others [13,28]stated that such prolonged delays can result in increase the risk of fluid and electrolyte depletion, sepsis and malnutrition. In our study, surgery resulted in closure of 12 fistulas (52.2%).

Mawdesley JE et al. [29] reported that for patients treated conservatively, a decreased probability of enterocutaneous fistula closure was associated with a high fistula output, co-morbidity and being referred from an external institutions while successful enterocutaneous fistula closure for patients treated surgically depends mainly to complexity of the fistula (presence of an internal abscess cavity and multiple fistulas).Sepsis, malnutrition, and fluid and electrolytes disturbances are the main causes of mortality.

In our study, the mortality rate was 21.7%.The mortality rate among patients treated conservatively was 4.3% and those who have had surgery were 17.3% (Table 5). Patients with small bowel, proximal high output fistulas have high mortality rates. Our findings are consistent with results of similar researches^.^ [9,10,13,16,30]Death due to duodenal and Jejunal fistulas have been reported to have double the death rates associated with distal ileal and colonic fistulas due to higher effluent [27]^,31^. Several variables used clinically to judge the likelihood of spontaneous closure are illustrated in Table 6. Our results showed that the main causes of failed conservative treatment of enterocutaneous fistulas are high output, proximal and complex fistulas associated with internal abscess cavities whereas the main causes of operative treatment were the underlying cause of the fistula such as malignancy, comorbidities, and overwhelming sepsis. Our findings were consistent with that of Mawdesley et al. [29] and Martinez ET al [30].

The main limitations of this study is that of any retrospective study, small number of patients involved and the follow-up periods were deemed insufficiently long to evaluate the management outcomes of various surgical repairs. Large sample-size and high-quality prospective studies with longer follow up period are required for better evaluation of the management outcomes of enterocutaneous fistulas.

## 5 Conclusion

Management of postoperative enterocutaneous fistulas is challenging and big surgical dilemma. Strict adherence to a standardized protocols and multidisciplinary approach can result in a better outcome. Initial conservative treatment is always required to optimize the general health of the patients by correcting nutritional status, replacement of fluid and electrolytes, improvement of serum albumin and transferrin and most importantly to control sepsis. Operative correction in a poor general condition and sepsis would be catastrophic for the patient. Surgery however is usually required for proximal, high output fistula and fistulas associated with diseased bowel. One -stage repair can be performed successfully in the majority of stable patients. The complexity of the fistulas, sepsis, high output effluent and comorbidities are the main factors affecting healing rates and fistula related mortality.

## Funds

None. Self-fund

## Ethical Approval

The study was approved by ethical committee of Basra medical college, University of Basra.

## Consent

Written informed consent was obtained from all the patients for publication of this case series.

## Author contribution

The surgical aspect of this study was done by dr. Ibrahim Falih Noori and the pulmonary function tests and changes in lungs volumes study was done by dr. Azza Sajid Jabbar.

## Registration of research studies

Name of the registry: www.researchregistry.com.

Unique identifying number or registration ID: researchregistry 6717.

Registration of research study: Registration ID: researchregistry 6717.

Hyperlink: https://www.researchregistry.com/browse-the-registry#home/.

## Guarantor

The authors are the sole guarantors for this work.

## Declaration of competing interest

Author declares no any conflicts of interest.
